# 303. The A-Stop Study: Rapid Tests for *Candida* Infection in Non-neutropenic Critically-ill Patients - A Multicentre Diagnostic Test Accuracy Study

**DOI:** 10.1093/ofid/ofae631.093

**Published:** 2025-01-29

**Authors:** Ronan McMullan

**Affiliations:** Queens University Belfast, Belfast, Northern Ireland, United Kingdom

## Abstract

**Background:**

Among critically-ill patients, antifungal therapy is usually driven by risk rather than a confirmed diagnosis. This leads to overtreatment, due to both low confidence in culture-based diagnostics and the time-critical nature of antifungal treatment. Such overtreatment undermines antimicrobial stewardship which could be improved if rapid, non-culture, tests reliably exclude disease.

**Methods:**

This was a prospective multicentre diagnostic test accuracy study of three rapid tests for Candida infection: beta-D-glucan (BDG), Associates of Cape Cod, and two PCR-based tests (Fungiplex Candida, Bruker Corporation; & T2Candida, T2 Biosystems). In brief, patients were eligible for recruitment within 24hr of being started empirically on antifungal therapy for suspected Candida infection if they were not neutropenic. In the primary analysis, diagnostic accuracy metrics were derived by comparing index test results to the EORTC/MSGERC definition of proven Candida disease for patients in intensive care units (ICU). In a secondary analysis, the tests were compared with a constructed reference standard for proven + probable Candida disease. Since the purpose of these tests was to rule-out disease, sensitivity and negative predictive value (NPV) were the focus.

**Results:**

Of 4005 patients screened for eligibility, 1251 participants were recruited from 57 UK ICUs between 2018-2023. Of these, 1223 were evaluable against the definition of proven disease and 54 met that definition. In total, 1215, 1217, and 507 participants had an evaluable BDG, Fungiplex, and T2Candida test, respectively. For proven disease, the most sensitive test was BDG (69%), followed by T2Candida (44%) and Fungiplex (28%); the NPV of each of these three tests was 97%. The sensitivity was predictably lower for the proven + probable disease group (N=124), ranging from 18%-58%, yet NPV remained above 90% for all three tests.

**Conclusion:**

There is an opportunity to reduce unnecessary antifungal prescribing in this patient group. Each of the tests evaluated had lower sensitivity than previously reported but, because of low disease prevalence, they had high NPV. Modelling the effects of using the tests to stop empiric treatment may further guide how best to apply the tests in practice.

**Disclosures:**

**All Authors**: No reported disclosures

